# Rheb1 promotes tumor progression through mTORC1 in MLL-AF9-initiated murine acute myeloid leukemia

**DOI:** 10.1186/s13045-016-0264-3

**Published:** 2016-04-12

**Authors:** Yanan Gao, Juan Gao, Minghao Li, Yawei Zheng, Yajie Wang, Hongyan Zhang, Weili Wang, Yajing Chu, Xiaomin Wang, Mingjiang Xu, Tao Cheng, Zhenyu Ju, Weiping Yuan

**Affiliations:** State Key Laboratory of Experimental Hematology, Institute of Hematology and Blood Diseases Hospital, Center for Stem Cell Medicine, CAMS & PUMC, Beijing, China; Department of Biochemistry and Molecular Biology, Miller School of Medicine, University of Miami, Miami, USA; Institute of Aging, Hangzhou Normal University, Hangzhou, 310036 China

**Keywords:** Rheb1, mTORC1, MLL-AF9, Leukemia, Rapamycin, 3BDO

## Abstract

**Background:**

The constitutive hyper-activation of phosphatidylinositol 3-kinase (PI3K)/protein kinase B (Akt)/mammalian target of rapamycin (mTOR) signaling pathways has frequently been associated with acute myeloid leukemia (AML). While many inhibitors targeting these pathways have been developed, the anti-leukemic effect was not as robust as expected. As part of the molecular link between PI3K/Akt and mTOR kinase, the role of Rheb1 in AML remains unexplored. Our study aims to explore the role of Rheb1 in AML and estimate whether Rheb1 could be a potential target of AML treatment.

**Methods:**

The expressions of *Rheb1* and other indicated genes were analyzed using real-time PCR. AML mouse model was established by retrovirus transduction. Leukemia cell properties and related signaling pathways were dissected by in vitro and in vivo studies. The transcriptional changes were analyzed via gene chip analysis. Molecular reagents including mTOR inhibitor and mTOR activator were used to evaluate the function of related signaling pathway in the mouse model.

**Results:**

We observed that Rheb1 is overexpressed in AML patients and the change of Rheb1 level in AML patients is associated with their median survival. Using a Rheb1-deficient MLL-AF9 murine AML model, we revealed that Rheb1 deletion prolonged the survival of AML mice by weakening LSC function. In addition, Rheb1 deletion arrested cell cycle progression and enhanced apoptosis of AML cells. Furthermore, while Rheb1 deletion reduced mTORC1 activity in AML cells, additional rapamycin treatment further decreased mTORC1 activity and increased the apoptosis of *Rheb1*^Δ/Δ^ AML cells. The mTOR activator 3BDO partially rescued mTORC1 signaling and inhibited apoptosis in *Rheb1*^Δ/Δ^ AML cells.

**Conclusions:**

Our data suggest that Rheb1 promotes AML progression through mTORC1 signaling pathway and combinational drug treatments targeting Rheb1 and mTOR might have a better therapeutic effect on leukemia.

**Electronic supplementary material:**

The online version of this article (doi:10.1186/s13045-016-0264-3) contains supplementary material, which is available to authorized users.

## Background

As the most common acute leukemia in adults, acute myeloid leukemia (AML) is a genetically heterogeneous neoplasm. Although this disease can initially be contained using classical chemotherapy, AML frequently relapses during the course of the disease [1]. The leukemia cells can be influenced by microenvironmental cues and also affect the normal niche of hematopoietic stem cells (HSCs) [2, 3]. A small fraction of AML cells can self-renew and are thus referred to as leukemic stem cells (LSCs). LSCs share properties with normal HSCs in sustaining normal hematopoietic hierarchy [4, 5]. LSCs have been implicated in the relapse of leukemia and are extensively studied as potential therapeutic targets. The signaling pathways that regulate the development and survival of these cells are of particular interest [5, 6].

Mammalian target of rapamycin (mTOR) is an evolutionarily conserved serine/threonine (Ser/Thr) kinase that senses and responds to multiple signals in eukaryotes. mTOR interacts with multi-proteins to form two distinct complexes, designated mTOR complex 1 (mTORC1) and mTOR complex 2 (mTORC2). mTORC1 primarily comprises mTOR, raptor, PRAS40, deptor, the Tti/Tel complex, and mLST8. This complex directly phosphorylates p70 ribosomal protein S6 kinase 1 (p70S6K1, phosphorylates ribosomal protein S6) and eukaryote translation initiation factor 4E binding protein 1 (4E-BP1) to regulate cell growth and survival [7]. Upon growth factor/cytokine stimulation, phosphatidylinositol 3-kinase (PI3K) is rapidly activated and recruits protein kinase B (Akt, PKB) to the plasma membrane. Akt is subsequently phosphorylated at its T308 and S473 sites. Following the phosphorylation of Akt or ERK1/2, TSC1/TSC2 is destabilized and the inhibition of mTOR signaling is relieved. The activation of the PI3K/Akt/mTOR signaling pathway has frequently been reported in AMLs [8, 9]. For example, a previous study showed that TSC1 deficiency disrupts the quiescence and long-term reconstitution of HSCs, reflecting over-activated mTORC1 [10]. The deletion of phosphatase and tensin homologue (PTEN), which negatively regulates the PI3K/Akt/mTOR pathway, leads to leukemia, including AML in mice [11]. In addition, the deletion of raptor, an essential component of mTORC1, suppresses AML progression through the enhanced apoptosis of AML cells [12]. Rapamycin, a natural mTOR kinase inhibitor, inhibits not only the generation but also the maintenance of leukemia in PTEN-evoked leukemogenesis [11]. Human AMLs have also been shown to respond to rapamycin [13], but with low efficacy, and new homologues of rapamycin (rapalogs) are being developed to treat AMLs [14].

Ras homologue enriched in the brain (Rheb) is an activator of mTORC1 [15, 16]. Two Rheb family members, Rheb1 (the original Rheb, hereafter referred to as Rheb1) and Rheb2 (also known as RhebL1), have been identified in mammals. Rheb1 is essential for murine development, as the loss of Rheb1 results in embryonic death at approximately midgestation [17], while Rheb2 is dispensable in mouse development [18]. The TSC complex, comprising TSC1, TSC2, and TBC1D7, are GTPase-activating proteins (GAPs) of Rheb [19–21]. Active Rheb might indirectly inhibit PI3K and mTORC2 signaling through the induction of various mTORC1-dependent negative feedback loops [22]. Previous studies have demonstrated that Rheb1 was overexpressed in various cancers, such as non-small cell lung cancer, liver cancer, bladder cancer, and prostate cancer [23–26]. The high level of Rheb1 was associated with the progression, metastasis, drug resistance, and poor prognosis of breast cancer patients [27]. The human cancer genome data analysis revealed recurrent mutations in Rheb1 [28]. In addition, Rheb1 overexpression also favors myc-mediated lymphomagenesis in a mouse model [29]. The findings indicate that Rheb1 contributes to tumorigenesis and might be a potential treatment target.

Although it has been clearly established that the dysregulation of mTORC1 promotes leukemogenesis, the role of Rheb1 in the development and maintenance of AML is not clear. In the present study, we examined the role of Rheb1 in the MLL-AF9 murine AML model and showed that Rheb1 deletion suppressed AML progression by impairing LSC function and reducing mTORC1 signaling.

## Results

### Rheb1 is overexpressed in human AMLs

**Fig. 1:**
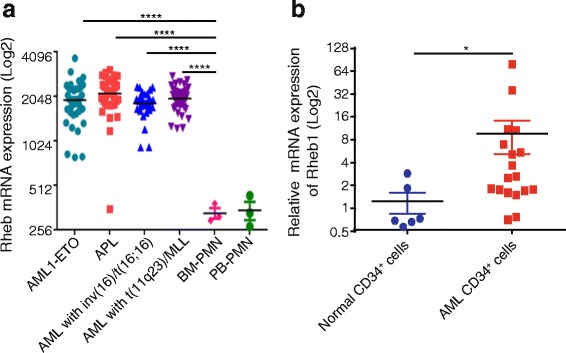
Rheb1 is overexpressed in human AML. **a** Rheb1 mRNA expression in human AML cells with t(8;21)(AMLI_ETO), t(15;17)(APL), inv(16)/t(16;16), and t(11q23)/MLL using the HemaExplorer database. **b** Rheb1 mRNA expression in human AML and normal CD34^+^ cells. The data were analyzed using the Mann-Whitney *U* test. The significance is indicated with *asterisks* (*P < 0.05; **P < 0.01; ***P < 0.001; ****P < 0.0001)

Previous studies have demonstrated that the PI3K/Akt/mTOR signaling pathway is involved in many cancers, including AMLs, thus implicating a role for Rheb1 in cancer progression/maintenance. Here, we used a curated database (the HemaExplorer, http://servers.binf.ku.dk/hemaexplorer), which provides gene expression profiles of processed mRNA, to analyze the mRNA expression level of Rheb1 in human AML. We observed that Rheb1 mRNA expression was substantially higher in human AMLs, such as AML with t(8;21)(AMLI_ETO), AML with t(15;17)(APL), AML with inv(16)/t(16;16), or AML with t(11q23)/MLL in contrast to normal polymorphonuclear cells (PMN) in the peripheral blood (PB) or bone marrow (BM) (Fig. 1a). Interestingly, Rheb1 mRNA overexpression was also confirmed in human AML CD34^+^ cells compared with normal CD34^+^ cells. We observed that Rheb1 mRNA expression was increased in human AML CD34^+^ cells compared with normal CD34^+^ cells (Fig. 1b). In addition, AML patients with higher Rheb1 expression showed reduced survival (median survival: 8.47 months) compared with patients with lower Rheb1 expression (median survival: 11.47 months) based on the Leukemia Gene Atlas (LGA) analysis (http://www.leukemia-gene-atlas.org/LGAtlas/LGAtlas.html#aboutUs) (Additional file 1: Figure S1A) [30]. We further accessed The Cancer Genome Atlas (TCGA) data via the cBioPortal for Cancer Genomics (http://www.cbioportal.org/public-portal) [31]. We found that AML patients with Rheb1 deletion showed increased survival (median survival: 18.96 months) compared with patients without Rheb1 deletion (median survival: 15.01 months) (Additional file 1: Figure S1B). These indicate that Rheb1 is a risk factor for AML.

### Rheb1 deletion suppresses AML progression in vivo

**Fig. 2:**
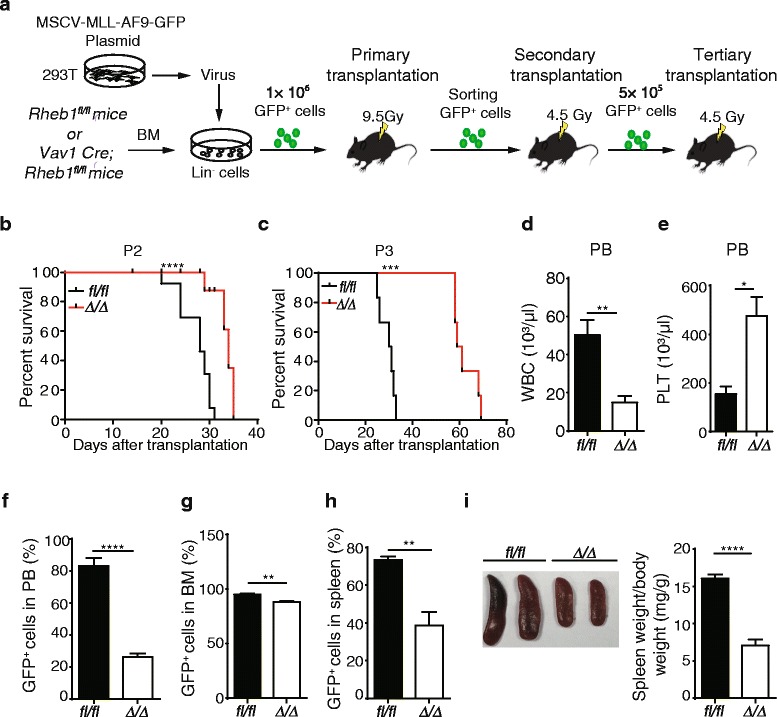
Rheb1 deletion prolongs the survival of AML mice. **a** Experimental scheme of the Rheb1-deficient MLL-AF9 mouse model. **b** The survival curve of *Rheb1* ^*fl/fl*^ and *Rheb1* ^Δ/Δ^ AML mice using Kaplan-Meier analysis (*n* = 11). Approximately 5 × 10^5^ P1 GFP^+^ cells from the two groups were transplanted into sublethally irradiated recipient mice (P2). **c** The survival curve of *Rheb1* ^*fl/fl*^ and *Rheb1* ^Δ/Δ^ AML mice calculated using Kaplan-Meier analysis (*n* = 6). Approximately 1 × 10^6^ P2 GFP^+^ cells obtained from the two groups were transplanted into sublethally irradiated recipient mice (P3). **d** The number of white blood cells (*WBC*) in PB (*n* = 4). **e** The number of platelets (*PLT*) in PB (*n* = 4). PB samples were obtained from AML mice when *Rheb1* ^*fl/fl*^ GFP^+^ cells in PB was approximately 80 %. **f**–**h** The percentage of GFP^+^ cells in the PB (**f**), BM (**g**), and spleen (**h**) of *Rheb1* ^*fl/fl*^ and *Rheb1* ^Δ/Δ^ AML mice by FACS analysis (*n* = 4). **i** The spleen size (*left*) and spleen weight to body weight ratio of *Rheb1* ^*fl/fl*^ and *Rheb1* ^Δ/Δ^ AML mice (*right*). All experiments were performed at least three times. The *values in the panels* represent the mean numbers ± SEM. *P < 0.05; **P < 0.01; ***P < 0.001; ****P < 0.0001

To assess the role of Rheb1 in AML, we first examined Rheb1 expression in the BM of wild-type (WT) mice and established MLL-AF9-induced AML mice by Western blotting analysis and found that Rheb1 protein was overexpressed in AML mice in comparison with WT mice (Additional file 1: Figure S2A). To further investigate the role of Rheb1 in murine AML, we first generated *Vav1-Cre*;*Rheb1*^*fl/fl*^ (*Rheb1*^Δ/Δ^) mice via crossing *Vav1-Cre* mice with *Rheb1*^*fl/fl*^ mice. Lineage-negative (Lin^−^) cells were isolated from *Rheb1*^*fl/fl*^ (control) or *Rheb1*^Δ/Δ^ mice and subsequently transduced with the MLL-AF9-GFP fusion gene. These genetically modified GFP^+^ cells were subsequently sorted and transplanted into lethally irradiated syngeneic recipients to generate MLL-AF9-induced *Rheb1*^*fl/fl*^ or *Rheb1*^Δ/Δ^ AML mice, respectively. The GFP^+^ cells were expanded three times in serial transplantations prior to analysis as depicted in Fig. 2a. Both recipient mice developed AML, and flow cytometric analysis showed that GFP^+^ cells were B220^−^ CD3^−^CD11b^+^ (Additional file 1: Figure S2B). The *Rheb1* gene was efficiently deleted in *Rheb1*^Δ/Δ^ GFP^+^ cells as demonstrated by RT-PCR analysis (Additional file 1: Figure S2C) and PCR (Additional file 1: Figure S2D).

To determine the role of Rheb1 in AML, we intravenously injected *Rheb1*^*fl/fl*^ or *Rheb1*^Δ/Δ^ GFP^+^ P2 or P3 cells (see “Methods” section) into sublethally irradiated recipient mice. We observed that Rheb1 deletion significantly prolonged the survival of AML mice compared with the control in both P2 and P3 groups. The survival of *Rheb1*^Δ/Δ^ GFP^+^ P3-recipient mice was much longer than that of P2-recipient mice, and the following experiments were performed using P3 cells in mice (Fig. 2b, c). Consistent with the less leukemia burden in *Rheb1*^Δ/Δ^ AML mice, the number of WBCs in the PB of *Rheb1*^Δ/Δ^ AML mice was significantly decreased, while the platelet (PLT) counts were increased compared with that of control AML mice (Fig. 2d, e). Decreased percentages of GFP^+^ cells in the PB, BM, and spleen were also evident in *Rheb1*^Δ/Δ^ AML mice in contrast with that in the controls (Fig. 2f–h). In addition, the spleen size in *Rheb1*^Δ/Δ^ AML mice was smaller, and the spleen weight (mg) to body weight (g) ratio was significantly decreased in *Rheb1*^Δ/Δ^ AML mice compared with the controls (Fig. 2i). Consistent with this finding, less AML cells were observed in histological section of the spleens obtained from *Rheb1*^Δ/Δ^ AML mice compared with the control (Additional file 1: Figure S2E), indicating less infiltration of *Rheb1*^Δ/Δ^ GFP^+^ cells in the spleen. Therefore, *Rheb1* deficiency significantly suppressed AML progression in vivo and prolonged the life span of AML mice.

## Rheb1 deficiency impairs LSC function

**Fig. 3:**
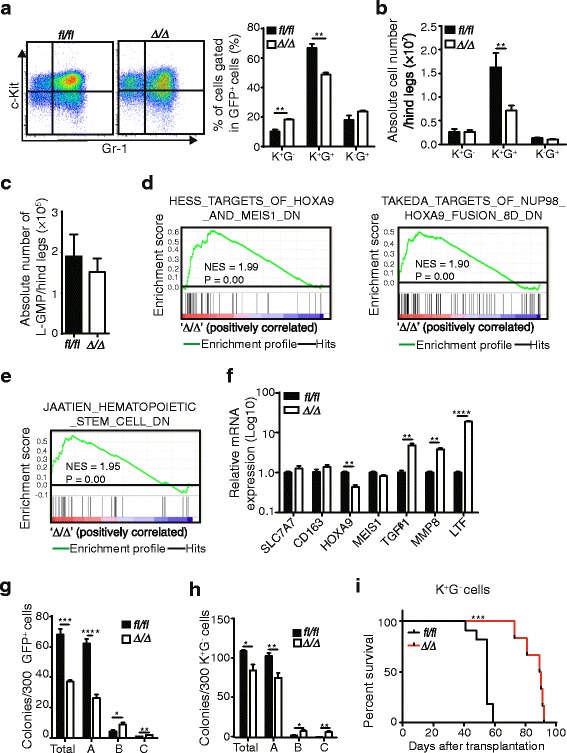
Rheb1 deletion impairs LSC function. **a** The differentiation profile of AML cells (*left*) and the percentages of subpopulations (*right*) using c-Kit and Gr-1 surface markers (*n* = 4). **b** The absolute number of these subpopulations in the BM (*n* = 4). **c** The absolute number of L-GMPs in the BM (*n* = 4). **d**, **e** The enrichment of selected gene targets for HOXA9 and MEIS1, NUP98-HOXA9 fusion genes (**d**), and HSC downregulation-related genes (**e**). The NES (normalized enrichment score) and *P* values are indicated in each plot. **f** The mRNA expression of the indicated genes assessed using RT-PCR. **g**, **h** Colony formation of GFP^+^ AML cells (**g**) and GFP^+^ K^+^G^−^ AML cells (**h**) that were sorted, replated in semisolid medium, and cultivated for 8 days prior to counting. The data show the mean colony numbers ± SEM. All experiments were performed at least three times. The data represent the mean numbers ± SEM. **i** The survival curve of *Rheb1* ^*fl/fl*^ and *Rheb1* ^Δ/Δ^ AML mice using Kaplan-Meier analysis (*n* = 6). Approximately 1000 P3 K^+^G^−^ cells from the two groups were transplanted into sub-lethally irradiated recipient mice (P4). *P < 0.05; **P < 0.01; ***P < 0.001; ****P < 0.0001

Previous studies using MLL-AF9 AML models have established that LSCs are enriched in c-Kit^+^Gr-1^−^ (K^+^G^−^) [6] or L-GMP populations [32]. To further delineate AML progression without Rheb1, the differentiation status of *Rheb1*^*fl/fl*^ or *Rheb1*^Δ/Δ^ AML cells was analyzed using the cell surface markers, c-Kit and Gr-1. We observed that the percentage of K^+^G^−^ cells was slightly increased, whereas the percentage of K^+^G^+^ cells was decreased, and the percentage of K^−^G^+^ cells was not different in the BM of *Rheb1*^Δ/Δ^ GFP^+^ cells compared with the control (Fig. 3a). Although the absolute cell number of K^+^G^−^ and K^−^G^+^ cells was not increased, the number of K^+^G^+^ cells in the BM was dramatically decreased in *Rheb1*-deficient AML cells (Fig. 3b). The L-GMP (Lin^low^ GFP^+^ c-Kit^+^ FcR-γ^+^ CD34^+^) frequency was subsequently analyzed, and no difference in the L-GMP frequency was observed between the two groups (Additional file 1: Figure S2F and Fig. 3c), consistent with the frequency of K^+^G^−^ cells (Fig. 3b). In addition, the percentages of L-GMP and K^+^G^−^ cells in the spleen were also not changed between the two groups (Additional file 1: Figure S2G and H). These results indicate that the Rheb1 deletion did not affect the absolute number of LSCs in the BM.

To determine whether the prolonged survival of *Rheb1*^Δ/Δ^ AML mice was due to impaired homing ability of AML cells, *Rheb1*^*fl/fl*^ or *Rheb1*^Δ/Δ^ GFP^+^ cells were transplanted into lethally irradiated recipient mice. The recipient mice were sacrificed at 24 h after transplantation, and the BM and spleen were analyzed for GFP^+^ AML cells. The absolute number of GFP^+^ cells homing to the BM and spleen was not changed between the two groups (Additional file 1: Figure S3A), indicating that homing is not a factor underlying the prolonged survival of *Rheb1*^Δ/Δ^ AML mice.

To explore the transcriptional landscape changes that distinguish *Rheb1*^*fl/fl*^ or *Rheb1*^Δ/Δ^ AML cells, gene expression microarray was performed with sorted *Rheb1*^*fl/fl*^ or *Rheb1*^Δ/Δ^ GFP^+^ cells. The deletion of Rheb1 in sorted *Rheb1*^Δ/Δ^ GFP^+^ cells was confirmed after measuring the mRNA expression levels in these cells using RT-PCR (data not shown). Gene set enrichment analysis (GSEA) [33] revealed the significant enrichment of downregulated genes in hematopoietic precursor cells expressing HOXA9 and MEIS1, CD34^+^ hematopoietic cells expressing the NUP98-HOXA9 fusion (Fig. 3d), and CD133^+^ hematopoietic stem cells (as an alternative for CD34^+^ cells) (Fig. 3e). The enriched and selected genes were further validated by RT-PCR. HOXA9, which is required for the survival of MLL-arranged acute leukemia, is overexpressed in approximately 50 % of AML and highly associated with poor prognosis [34, 35]. Here, we observed that the HOXA9 expression level decreased due to Rheb1 deletion (Fig. 3f). The mRNA expressions of TGFβ1, MMP8 (matrix metalloproteinase 8), and LTF (lactoferrin), which all show tumor suppressing ability [36–38], were significantly increased in *Rheb1*^Δ/Δ^ GFP^+^ cells compared with the control (Fig. 3f). This result suggests that Rheb1 deletion contributes to AML progression through the downregulation of HOXA9 expression and the upregulation of tumor suppressor gene expression, which potentially affects LSC function. To further investigate the effect of Rheb1 deletion on LSC function, we examined the colony-forming ability of *Rheb1*^*fl/fl*^ and *Rheb1*^Δ/Δ^ AML cells to characterize the progression and stemness of AML cells. The sorted GFP^+^ or K^+^G^−^ cells were cultured in methylcellulose medium supplemented with IL-3, IL-6, SCF, and GM-CSF, and the colonies were counted after 7 to 8 days of culture. A previous study established that colonies show progression in AML cell maturation from type A to type B to type C, demonstrating a progressive loss of stemness [39]. Here, we observed that the number of total colonies was significantly decreased in *Rheb1*^Δ/Δ^ GFP^+^ cells compared with the control. Interestingly, the number of type A colonies was reduced, while the number of type B and type C colonies was increased (Fig. 3g). Similar observations were obtained with *Rheb1*^Δ/Δ^ K^+^G^−^ cells compared with the control (Fig. 3h). Furthermore, K^+^G^−^ cell transplantation showed that the survival of *Rheb1*^Δ/Δ^ AML mice was significantly prolonged than the control mice (Fig. 3i). These results supported that the loss of Rheb1 impaired AML progression and affected the stemness of AML cells.

### Rheb1 deficiency arrests more AML cells in the G0 phase

**Fig. 4:**
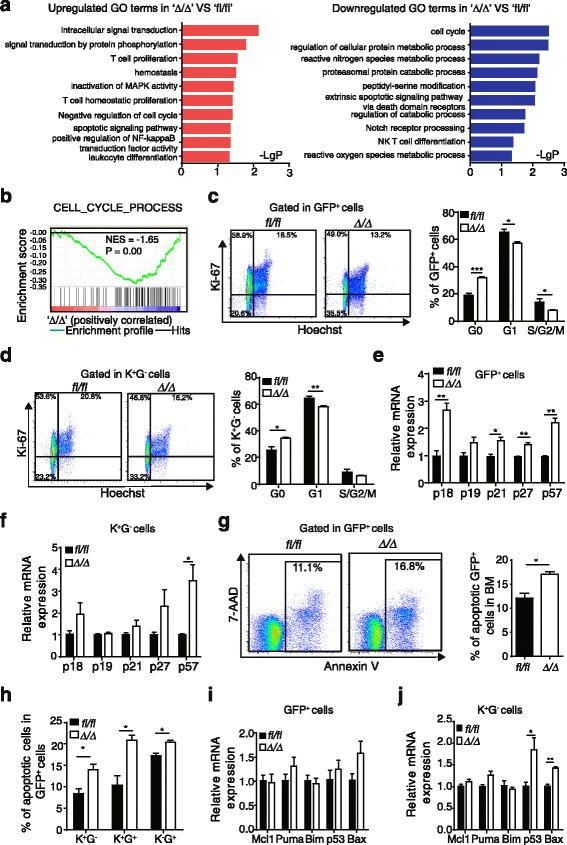
Rheb1 deletion arrests cell cycle progression and increases the apoptosis of AML cells. **a** Upregulated (*left*) and downregulated (*right*) GO terms of differentially expressed genes in *Rheb1* ^Δ/Δ^ GFP^+^ cells vs. the WT control. **b** Enrichment of the cell cycle process. NES (normalized enrichment score) and *P* values are indicated in each plot. **c**, **d** The cell cycle status of GFP^+^ (**c**) and K^+^G^−^ cells (**d**), *n* = 4. **e**, **f** GFP^+^ cells (**e**) and K^+^G^−^ cells (**f**) were sorted, and their RNAs were isolated for indicated gene analysis using RT-PCR. The data represent the mean numbers ± SEM. **g**, **h** The apoptosis status of GFP^+^ (**g**) and K^+^G^−^ cells (**h**), *n* = 4. **i**, **j** GFP^+^ cells (**i**) and K^+^G^−^ cells (**j**) were sorted, and RNAs were isolated and analyzed using RT-PCR for the indicated genes. The data represent the mean numbers ± SEM. *P < 0.05; **P < 0.01; ***P < 0.001

To explore the mechanism by which Rheb1 promotes AML progression, we performed gene ontology (GO) enrichment analysis of the 1487 differentially expressed genes derived from the microarray expression data (Additional file 1: Figure S3B). The analysis indicated that “negative regulation of cell cycle” and “apoptotic signaling pathway” categories were upregulated, while the “cell cycle” category was downregulated (Fig. 4a).

**Fig. 5:**
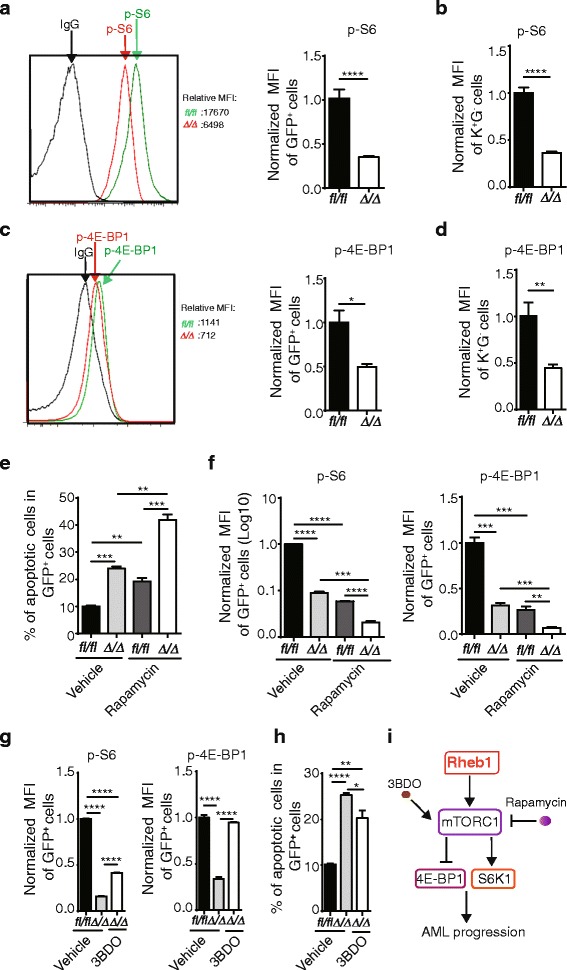
Rheb1 deletion reduces mTORC1 signaling in AML cells. **a** FACS analysis of p-S6 in GFP^+^ cells. GFP^+^ cells were sorted from fresh BM cells. The *left panel* shows the mean fluorescent intensity (MFI) of both groups, the *right panel* shows the normalized MFI of these groups (*n* = 4). IgG was used as an internal control. **b** The normalized MFI of p-S6 in the LSC cells of the two groups (*n* = 4). **c** FACS analysis of p-4E-BP1 in GFP^+^ cells. GFP^+^ cells were sorted from fresh BM cells. The *left panel* shows the mean fluorescent intensity (MFI) of both groups, and the *right panel* shows the normalized MFI of these groups (*n* = 4). IgG was used as an internal control. **d** The normalized MFI of p-4E-BP1 in the LSC cells of the two groups (*n* = 4). **e** The percentage of apoptotic GFP^+^ cells treated with mock treatment (absolute ethanol without rapamycin, vehicle), and rapamycin was dissolved in absolute ethanol (EtOH) (*n* = 3). The data represent the mean numbers ± SEM. **f** The normalized MFI (Log10) of p-S6 in cultured GFP^+^ cells of the two groups treated with EtOH without rapamycin (*n* = 3). **g** The normalized MFI (Log10) of p-S6 in cultured GFP^+^ cells of the two groups treated with DMSO without 3BDO (*n* = 3). **h** The percentage of apoptotic GFP^+^ cells treated with mock treatment (DMSO without 3BDO, vehicle), and 3BDO was dissolved in DMSO (*n* = 3). **i** The Rheb1/mTORC1 signaling pathway in AML progression. *P < 0.05; **P < 0.01; ***P < 0.001; ****P < 0.0001

Because cancer progression is determined by the rate of cell proliferation and death [40], GSEA was used to analyze cell cycle and apoptosis-related gene sets. We observed that genes associated with cell cycle processes (Fig. 4b) and the M phase of the mitotic cell cycle (Additional file 1: Figure S3C) were enriched in control AML cells. Flow cytometric analysis of the cell cycle status of fresh BM GFP^+^ cells using Ki-67 and Hoechst 33342 staining revealed an increased proportion of cells in the G0 phase and a decreased proportion of cells in the G1 and S/G2/M phases of *Rheb1*^Δ/Δ^ GFP^+^ cells and its corresponding subpopulations (K^+^G^−^ cells and K^+^G^+^ cells) (Fig. 4c, d and Additional file 1: Figure S3D). Interestingly, the Rheb1 deletion had no effect on the cell cycle status of K^−^G^+^ cells (Additional file 1: Figure S3E). Since cell cycle progression is controlled through various regulators, such as cyclins, cyclin-dependent kinases (CDKs) [41], and CDK inhibitors (CKIs) [42], we also examined the expression of these genes and observed that the mRNA expressions of p18, p21, p27, and p57 were increased in *Rheb1*^Δ/Δ^ GFP^+^ cells compared with control (Fig. 4e), while the mRNA expression of cyclin G2, a negative regulator of cell cycle progression [43], was also upregulated in *Rheb1*^Δ/Δ^ GFP^+^ cells compared with control (Additional file 1: Figure S3F). These findings suggest that Rheb1 might promote AML cell cycle progression through the inhibition of the transcription of CKIs and cyclin G2. Furthermore, in *Rheb1*^Δ/Δ^ K^+^G^−^ cells, the mRNA expression of p57 was upregulated compared with control (Fig. 5e and Additional file 1: Figure S3G). Therefore, Rheb1 promotes LSC cell cycle progression probably via the inhibition of p57 transcription.

### Rheb1 deletion induces the apoptosis of LSC cells

BM GFP^+^ cells were sorted and cultured, and the apoptosis status was examined at 72 h after culture through flow cytometry using 7-AAD and annexin V. The percentage of apoptotic GFP^+^ cells (annexin V^+^) in the BM was increased (Fig. 4g), and the proportions of apoptotic K^+^G^−^, K^+^G^+^, and K^−^G^+^ cells (annexin V^+^) were also significantly increased in Rheb1-deficient AML cells compared with the control (Fig. 4h). Apoptosis is regulated by anti-apoptotic signals, including Mcl-1, and pro-apoptotic signals, including Puma, Bim, Bax, and Bak [44]. While the p53 tumor suppressor inhibits cell growth through both the inhibition of cell cycle progression and the activation of apoptosis, the latter is considered to be essential for the tumor suppressor ability of this protein [45]. To explore the potential cause of apoptosis changes, we examined the mRNA expression of these genes. Although we observed no obvious difference in the mRNA expression in *Rheb1*^Δ/Δ^ and WT GFP^+^ cells (Fig. 4i), the mRNA expression of p53 and Bax was significantly increased in *Rheb1*^Δ/Δ^ K^+^G^−^ cells compared with the WT control (Fig. 4j), suggesting that Rheb1 inhibits the apoptosis of AML LSC cells through the repression of the mRNA expression of p53 and Bax.

### Rheb1 deletion suppresses AML progression via mTORC1 signaling pathway

Because Rheb1 directly activates mTORC1, the mTORC1 function was evaluated based on the phosphorylation levels of its downstream targets (S6 and 4E-BP1) using flow cytometry in *Rheb1*^Δ/Δ^ AML cells. As expected, the phosphorylation levels of S6 and 4E-BP1 in GFP^+^ cells and corresponding subpopulations (including K^+^G^−^, K^+^G^+^, and K^−^G^+^ cells) were significantly decreased in *Rheb1*^Δ/Δ^ AML cells compared with the control (Fig. 5a–d and Additional file 1: Figure S3H and I). These findings indicate that the reduced progression of AML in *Rheb1*^Δ/Δ^ cells might be attributed to attenuated mTORC1 activity.

We subsequently analyzed the HemaExplorer database and observed that mTOR mRNA was indeed overexpressed in AML with inv(16)/t(16;16) and AML with t(11q23)/MLL compared with PMN in the PB and BM (Additional file 1: Figure S4A). In addition, the 4E-BP1 mRNA was also overexpressed in these AMLs, although the S6 mRNA expression level in AML was not available from the datasets (Additional file 1: Figure S4B). The consistency of the overexpression of Rheb1, mTOR, and 4E-BP1 mRNA in these AML suggests that AML progression is clinically associated with Rheb1-regulated mTORC1 signaling. Interestingly, mTOR was not all overexpressed in the AML types in these datasets, suggesting the potential involvement of other signaling pathway(s) in AML (Additional file 1: Figure S4A). In addition, phagosome and Jak-stat signaling pathways were enriched in upregulated pathways in the KEGG analysis (Additional file 1: Figure S4C) (http://www.genome.jp/kegg/), while the p53 signaling pathway and cell cycle were enriched in downregulated pathways in KEGG analysis (Additional file 1: Figure S4D).

To further evaluate the extent of how Rheb1 regulates AML progression through mTORC1 signaling, BM GFP^+^ cells were sorted and cultured for 24 h prior to rapamycin addition. At 48 h after rapamycin treatment, the apoptosis status of GFP^+^ cells and the S6 and 4E-BP1 phosphorylation levels were assessed by flow cytometry. We observed that Rheb1-deficient GFP^+^ cells have increased apoptosis rates under the mock treatment (no rapamycin, with ethanol only, will also be referred as the control), consistent with the findings in Fig. 4g. Significantly, the apoptosis of both *Rheb1*^*fl/fl*^ and *Rheb1*^Δ/Δ^ GFP^+^ cells was enhanced under rapamycin treatment (Fig. 5e). To investigate whether the increased apoptosis of AML cells reflected mTORC1 inhibition through rapamycin, the S6 and 4E-BP1 phosphorylation levels were examined. We observed that the phosphorylation levels of S6 and 4E-BP1 in *Rheb1*^Δ/Δ^ GFP^+^ cells were lower than in *Rheb1*^*fl/fl*^ GFP^+^ cells under the control treatment (Fig. 5f), consistent with the findings shown in Fig. 5a. Rapamycin treatment decreased both S6 and 4E-BP1 phosphorylation levels in both *Rheb1*^*fl/fl*^ and *Rheb1*^Δ/Δ^ GFP^+^ cells compared with their corresponding controls, respectively (Fig. 5f). These findings indicated that mTORC1 protected AML cells from apoptosis and Rheb1 inhibited AML cells apoptosis through mTORC1. Interestingly, the phosphorylation level of S6 and 4E-BP1 in *Rheb1*^Δ/Δ^ GFP^+^ cells were further decreased after rapamycin treatment compared with the control (Fig. 5f), indicating that Rheb1 is partially responsible for mTORC1 signaling in AML cells.

3-Benzyl-5-((2-nitrophenoxy) methyl)-dihydrofuran-2(3H)-one (3BDO), a small molecular compound, has been demonstrated to be a novel mTOR activator that can significantly increase the phosphorylation of RPS6KB1 and EIF4EBP1 in HUVECs [46]. To examine how leukemia cells response to the rescued mTORC1 signaling, BM GFP^+^ cells (with or without Rheb1) were sorted and cultured for 24 h prior to 3BDO addition. At 48 h after 3BDO treatment, the S6 and 4E-BP1 phosphorylation levels and the apoptosis status of GFP^+^ cells were assessed by flow cytometry. We observed that 3BDO increased the S6 and 4E-BP1 phosphorylation of *Rheb1*^Δ/Δ^ GFP^+^ cells (Fig. 5g). The apoptosis of *Rheb1*^Δ/Δ^ GFP^+^ cells was reduced significantly under 3BDO treatment in comparison to *Rheb1*^Δ/Δ^ GFP^+^ cells with vehicle (DMSO) treatment but not to the same level as *Rheb1*^*fl/fl*^ GFP^+^ cells with vehicle treatment (Fig. 5h), indicating a partial reversal of increased apoptosis due to loss of Rheb1.

## Discussion

Rheb1 has been demonstrated as a molecular link between upstream PI3K/Akt signaling and downstream mTOR kinase to regulate cell growth [16, 47]. The PI3K/Akt/mTOR signaling pathway has been demonstrated to play numerous vital roles in cell survival and cell metabolism [48, 49]. The constitutive activation of PI3K/Akt/mTOR signaling was observed in 50–80 % of AML patients and has been associated with poor prognosis [50, 51]. Many inhibitors targeting this signaling pathway, either alone or in combination, have been developed, but with mediocre anti-leukemic efficacy [52]. Although Rheb1 has been shown to be mutated in cancer [28], the role of Rheb1 in AML remains unexplored. Here, we observed that in human AMLs, Rheb1, and mTOR mRNA were overexpressed (Fig. 1a and Additional file 1: Figure S4A). Using a Rheb1-deficient MLL-AF9 murine leukemia model, we further demonstrated that Rheb1 positively regulates leukemic cell growth via mTORC1 (Fig. 2b).

LSCs are composed of a minor subset of AML cells that are responsible for leukemia initiation, progression, and relapse [53]. LSCs are frequently insensitive to chemotherapy and therefore considered potential therapeutic targets for the eradication of cancer [54]. In the present study, the Rheb1 deletion did not change the LSC number in mouse BM, but the lifespan of AML mice was significantly prolonged. Additional experiments revealed that more Rheb1-deficient AML cells were arrested in the G0 phase with several upregulated CKIs. GSEA showed the enrichment of downregulated genes in hematopoietic progenitor or stem cells in *Rheb1*^Δ/Δ^ GFP^+^ cells, and some of these selected genes were confirmed by RT-PCR (Fig. 3d–f). In addition, *Rheb1*^Δ/Δ^ LSCs showed reduced colony formation, and the colonies exhibited reduced stemness in vitro (Fig. 3g, h). Furthermore, LSC transplantation showed Rheb1 deletion significantly prolonged the survival of AML mice in vivo (Fig. 3i). Thus, Rheb1 is required for LSC functional maintenance and AML progression. LSCs are known to resemble HSCs that give rise to all lineages of blood cells [55]. Interestingly, we found that under steady condition, Rheb1 deletion in hematopoietic system impaired HSC self-renewal and differentiation capability (data not shown) as in *Rheb1*^Δ/Δ^ LSCs.

Apoptosis is considered a key process for cancer progression or remission. Although apoptotic signaling circuitry in mammals is complicated and redundant with multiple regulators and effectors, this process offers attractive novel therapeutic targets along the pathway [40]. Here, we showed that the Rheb1 deletion increased the proportion of apoptotic AML cells, including LSCs, coincident with the upregulation of p53 and Bax mRNA expression (Fig. 4g–j). The enhanced apoptosis of *Rheb1*^Δ/Δ^ LSCs was further confirmed based on the observation that GFP^+^ cells and LSCs showed reduced p-4E-BP1, indicating the induction of apoptosis in tumor cells [56] and suggesting that Rheb1 might be a potential drug target for LSCs.

As a direct downstream effector of Rheb1, mTORC1 is essential for cell growth and metabolism. The deletion of raptor, a core component of mTORC1 [57], in the MLL-AF9 murine leukemia model, inhibited mTORC1 activity as evidenced by the decreased phosphorylation of S6 and 4E-BP1, decreased proliferation, and enhanced apoptosis of AML cells. These resulted in the prolonged survival of raptor-deficient AML mice, although the LSC number was increased in the raptor-deficient AML mouse model [12]. The discrepancy in LSC numbers in Rheb1- and raptor-deficient AML mouse models likely reflects the extent of mTORC1 inhibition. Indeed, we found that rapamycin treatment further decreased the phosphorylation level of S6 and 4E-BP1 and enhanced apoptosis in *Rheb1*^Δ/Δ^ GFP^+^ cells (Fig. 5e, f), while mTOR activator 3BDO increased the mTORC1 signal, partially rescued the augmented apoptosis of *Rheb1*^Δ/Δ^ GFP^+^ cells (Fig. 5g, h). Additionally, microarray analysis also showed that several mTORC1-independent pathways were also significantly altered (Additional file 1: Figure S4C and D). All these suggest the involvement of either a non-canonical Rheb1 pathway or the indirect effects resulting from the loss of Rheb1 [58, 59].

Despite advances in the current understanding of AML, the development of clinically effective therapies to eradicate AMLs has shown limited success, likely reflecting the existence of LSCs. Here, we convincingly showed that rapamycin could further enhance apoptosis in Rheb-deficient AML or LSC cells, suggesting the combinational use of Rheb1-specific inhibitors and improved rapalogs that better target mTORC1 might eradicate LSCs in AML patients with higher Rheb1 or mTORC1 levels.

## Conclusions

Rheb1 is overexpressed in human AML. Rheb1 deletion prolongs the survival of AML mice by reducing mTORC1 signaling and increasing apoptosis in AML cells. Rapamycin could further reduce mTORC1 signaling and increase the apoptosis of *Rheb1*^Δ/Δ^ GFP^+^ cells, while 3BDO partially rescues the apoptosis of *Rheb1*^Δ/Δ^ GFP^+^ cells through the activation of mTORC1 pathway (Fig. 5i). Our study thus suggests that combinational therapy targeting both Rheb1 and mTORC1 might be more efficient in eradicating leukemia.

## Methods

### Mice and genotyping

*Rheb1*^*fl/fl*^ mice were a kind gift from Dr. Bo Xiao [18]. Transgenic mice expressing Cre recombinase under the control of the *Vav1* promoter (*Vav1 Cre*) were purchased from the Jackson Lab. The *Rheb1*^*fl/fl*^ mice were crossed with *Vav1-Cre* mice to generate the specific deletion of Rheb1 in the hematopoietic system. The *Vav1-Cre*;*Rheb1*^*fl/fl*^ (*Rheb1*^Δ/Δ^) genotype was determined using PCR with the following primers to identify *Rheb1*^*fl/fl*^ or *Rheb1*^Δ/Δ^ mice: *Rheb1*, 5′-GCC CAG AAC ATC TGT TCC AT-3′ and 5′-GGT ACC CAC AAC CTG ACA CC-3′; *Vav1-Cre*, 5′-AGATGCCAGGACATCAGGAACCTG-3′ and 5′-ATCAGCCACACCAGACACAGAGATC-3′. All animal protocols were approved by the Institutional Animal Care and Use Committee (IACUC), Institute of Hematology and Blood Diseases Hospital, CAMS/PUMC. All surgeries were performed under sodium pentobarbital anesthesia, and all efforts were made to minimize mouse suffering.

### Human specimens

Normal human BM mononuclear cells (BM-MNCs) were obtained from allogeneic transplantation donors at the Institute of Hematology and Blood Diseases Hospital, CAMS/PUMC. Primary human BM AML cells were obtained from State Key Laboratory of Experimental Hematology, Institute of Hematology and Blood Diseases Hospital, CAMS/PUMC. Specimen acquisition was approved by Institutional Review Boards (IRB), State Key Laboratory of Experimental Hematology, Institute of Hematology and Blood Diseases Hospital, CAMS/PUMC. All donors signed the informed consent forms. The CD34^+^ cells were enriched using a CD34 Microbead Kit (Miltenyi, German).

### Establishment of the MLL-AF9 AML mouse model

MSCV-MLL-AF9-GFP plasmids were transduced into 293T cells to produce MLL-AF9 retroviruses using Lipofectamine 2000 (Invitrogen). Lineage negative (Lin^−^) cells were isolated from *Rheb1*^*fl/fl*^ or *Rheb1*^Δ/Δ^ mice and infected with MLL-AF9 retroviruses. At 72 h after infection, GFP^+^ cells were sorted and intravenously injected into lethally irradiated C57BL/6J-recipient mice. The GFP^+^ cells of primary AML mice were obtained as P0 cells. P0 cells were intravenously injected into secondary (P1) sub-lethally irradiated C57BL/6J-recipient mice to expand leukemic cells until P2 generation and subsequently to P3 generation. All subsequent experiments were performed using P3 cells.

### Flow cytometry

Peripheral blood (PB) was obtained from either the tail veins or retro-orbital bleeding of mice, and red blood cells (RBCs) were lysed using ammonium chloride-potassium bicarbonate buffer prior to staining. Bone marrow (BM) cells were flushed out from the tibias, femurs, and ilia using a 25-gauge needle with PBS supplemented with 2 % fetal bovine serum (FBS) and 20 mM EDTA (abbreviated as PBE). The spleen cells were suspended as a single cell suspension. The cells were stained with antibodies purchased from either eBioscience (San Diego, CA, USA) or BD Bioscience (New Jersey, NJ, USA). For the analysis of intracellular proteins, 1 × 10^6^ GFP^+^ cells were sorted from fresh BM cells. The sorted GFP^+^ cells were labeled with surface antibodies, fixed with 4 % paraformaldehyde, and permeabilized with 0.1 % Triton X100, and subsequently washed twice with 1 ml of cold PBE, resuspended with cold PBS supplemented with 25 % FBS, and intracellularly stained with antibodies against p-S6(Ser240/244) and p-4EBP1(Thr37/46). The cells were analyzed using flow cytometry. FlowJo software was used to analyze the results.

For cell cycle analysis, fresh BM GFP^+^ cells were sorted, labeled with surface antibodies, fixed and permeabilized with BD IntraSure Kit (BD Bioscience), and then intracellularly stained with antibodies against Ki67 (BD Bioscience) and Hoechst 33342 (Sigma-Aldrich). For the apoptosis assay, BM GFP^+^ cells were sorted from leukemic mice, cultured for 72 h in vitro and labeled with surface antibodies, fixed and permeabilized using the BD IntraSure Kit (BD Bioscience), and subsequently intracellularly stained with annexin V and 7-AAD (BD Bioscience).

### Mouse colony-forming cell assay (CFC)

GFP^+^ cells were sorted and cultured in methylcellulose medium (catalog #03231, StemCell Technologies) containing 50 ng/ml mSCF, 10 ng/ml mIL-6, 10 ng/ml mIL-3, and 10 ng/ml mGM-CSF (all from Peprotech). The sorted cells were cultured in 24-well plates at a volume of 0.5 ml, with 300 cells. The colonies were counted after 7–10 days of incubation.

### Cell culture

BM GFP^+^ cells were sorted and cultured in IMDM (Gibco) supplemented with 10 % FBS and 50 ng/ml mSCF, 10 ng/ml mIL-6, and 10 ng/ml mIL-3 (all from Peprotech, USA) for 24 h. The sorted cells were cultured in 24-well plates at a volume of 1.0 ml, with 4 × 10^4^ cells. After 24 h of culture, rapamycin (LC Lab, USA) was added at a final concentration of 5.45 nM/ml or 3BDO (a kind gift from Dr. Junying Miao) was added to the medium containing GFP^+^ cells at a final concentration of 30 nM/ml. The same volume of absolute ethanol or DMSO was added to the control group (vehicle group). At 48 h after drug treatment, the cells were collected, and the apoptotic status, and p-S6 and p-4E-BP1 levels were analyzed through flow cytometry.

### Microarray analysis and human AML database

GFP^+^ cells were sorted using a BD FACS Aria III flow cytometer, and RNAs were extracted with TRIzol reagent (Invitrogen, Canada) and purified using the RNeasy Mini Kit (Qiagen, German). Affymetrix GeneChip® Mouse Transcriptome Assay 1.0 was performed to estimate the whole transcriptome expression changes. The differentially expressed genes were filtered as *P* < 0.05 and fold change >1.2. The gene-set enrichment data were analyzed using GSEA, GO, and KEGG (FDR < 0.25, *P* < 0.05). The HemaExplorer (http://servers.binf.ku.dk/hemaexplorer) is a curated database that provides the gene expression profiles of processed mRNA from Human AMLs. We obtained the mRNA expression data from the database, and the raw data were photographed using GraphPad Prism 6.0.

### Real-time PCR (RT-PCR)

Total RNA from GFP^+^ cells was extracted using the RNeasy Mini Kit (Qiagen). First-strand cDNA was synthesized with oligo (dT) primer according to the manufacturer’s instructions. The number of transgene copies in the individual samples was determined by RT-PCR (Stepone Fast Real-Time PCR system; Applied Biosystems) using FastStart Universal SYBR Green PCR Master mix (Roche). All primer sequences are listed in Additional file 1: Table S1.

### Statistical analysis

GraphPad Prism 6.0 and SPSS 21.0 statistical software were used for statistical analyses. Kaplan-Maier survival curves were constructed using GraphPad Prism 6.0, and the log-rank (Mantel-Cox) analysis was used to analyze the results. For bar graphs, the unpaired two-tailed Student’s *t* test was used to compute the *P* values. *P* < 0.05 was considered significant. Significant differences are indicated with asterisks (*P < 0.05; **P < 0.01; ***P < 0.001; ****P < 0.0001).

## Additional file

Additional file 1: Figure S1. The correlation of Rheb1 expression level with AML prognosis. (A) Kaplan-Meier event-free survival curves of AML patients, with low (≤50 % quantile, *n* = 426) and high (>50 % quantile, n = 3) expression based on Verhaak et al.’s dataset. (B) Kaplan-Meier overall survival curves of AML patients, w/o Rheb1 deletion based on the cBioPortal database. Figure S2. The genotype of established AML mouse model. (A) Western blotting of Rheb1 protein level in WT and AML cells (*n* = 3). (B) Flow cytometric analysis of AML cells. (C) Rheb1 mRNA expression of AML cells. (D) PCR genotype of AML mice. (E) Histopathological analysis of the spleens in AML mice. (F) Gating strategy for L-GMPs in the spleen. (G) L-GMP frequency in the spleen (*n* = 4). (H) The percentages of AML subpopulations in the spleen (*n* = 4). The data are the mean numbers ± SEM. Figure S3. The function and related signaling pathway of AML cells. (A) GFP^+^ cells homing to the BM (*left panel*) and spleen (*right panel*), *n* = 5. (B) The differentially expressed genes of AML cells. (C) Enrichment of M phase mitotic cell cycle-related genes in GFP^+^ cells. (D and E) The cell cycle status of K^+^G^+^ (D) and K^−^G^+^ cells (E) (*n* = 4). (F and G) The mRNA levels of the indicated genes in GFP^+^ (F) and K^+^G^−^ cells (G). The data are the mean numbers ± SEM. (H and I) The normalized MFI of p-S6 (H) and p-4E-BP1 (I) of K^+^G^+^ and K^−^G^+^ cells in the BM (*n* = 4). The data are the mean numbers ± SEM. Figure S4. The signaling pathway in human and murine AML. (A and B) mTOR (A) and 4E-BP1 (B) mRNA expression of human AML cells in the HemaExplorer database. (C and D) KEGG analysis of the upregulated (C) and downregulated (D) pathways in *Rheb1* ^*Δ/Δ*^ vs *Rheb1* ^*fl/fl*^ GFP^+^ cells. Table S1. Primer sequence list. (PDF 30124 kb)
