# Supplementary material for: Rheb1 promotes tumor progression through mTORC1 in MLL-AF9-initiated murine acute myeloid leukemia
Source: J Hematol Oncol. 2016 Apr 12;9:36. doi: 10.1186/s13045-016-0264-3 (PMC4830070; doi:10.1186/s13045-016-0264-3)
Supplement: Additional file 1: Figure S1. — The correlation of Rheb1 expression level with AML prognosis. (A) Kaplan-Meier event-free survival curves of AML patients, with low (≤50 % quantile, n = 426) and high (>50 % quantile, n = 3) expression based on Verhaak et al.’s dataset. (B) Kaplan-Meier overall survival curves of AML patients, w/o Rheb1 deletion based on the cBioPortal database. Figure S2. The genotype of established AML mouse model. (A) Western blotting of Rheb1 protein level in WT and AML cells (n = 3). (B) Flow cytometric analysis of AML cells. (C) Rheb1 mRNA expression of AML cells. (D) PCR genotype of AML mice. (E) Histopathological analysis of the spleens in AML mice. (F) Gating strategy for L-GMPs in the spleen. (G) L-GMP frequency in the spleen (n = 4). (H) The percentages of AML subpopulations in the spleen (n = 4). The data are the mean numbers ± SEM. Figure S3. The function and related signaling pathway of AML cells. (A) GFP+ cells homing to the BM (left panel) and spleen (right panel), n = 5. (B) The differentially expressed genes of AML cells. (C) Enrichment of M phase mitotic cell cycle-related genes in GFP+ cells. (D and E) The cell cycle status of K+G+ (D) and K−G+ cells (E) (n = 4). (F and G) The mRNA levels of the indicated genes in GFP+ (F) and K+G− cells (G). The data are the mean numbers ± SEM. (H and I) The normalized MFI of p-S6 (H) and p-4E-BP1 (I) of K+G+ and K−G+ cells in the BM (n = 4). The data are the mean numbers ± SEM. Figure S4. The signaling pathway in human and murine AML. (A and B) mTOR (A) and 4E-BP1 (B) mRNA expression of human AML cells in the HemaExplorer database. (C and D) KEGG analysis of the upregulated (C) and downregulated (D) pathways in Rheb1 Δ/Δ vs Rheb1 fl/fl GFP+ cells. Table S1. Primer sequence list. (PDF 30124 kb) [file 13045_2016_264_MOESM1_ESM.pdf]

SUPPLEMENTARY FIGURE LEGENDS

Figure S1 The correlation of Rheb1 expression level with AML prognosis. (A) Kaplan-Meier curves of event-free survival of patients with AML, with low expression (≤ 50% quantile, n = 426) and high expression (> 50% quantile, n = 3) based on Verhaak *et al*.'s dataset. (B) Kaplan-Meier curves of overall survival of patients with AML, with or without Rheb1 deletion based on TCGA data in the cBioportal database.

Figure S2 The genotype of established AML mouse model. (A) Western blotting analysis of Rheb1 protein level in WT and AML cells and their densitometry value using β-actin as an internal control (n = 3). (B) Flow cytometric analysis of GFP^+ AML cells using CD3, B220 and CD11b. (C) Rheb1 mRNA expression of *Rheb1*$^{fl/fl}$ and *Rheb1*$^{Δ/Δ}$ AML cells. (D) PCR confirmation of the genotype of mice. (E) Histopathological analysis of spleen sections in *Rheb1*$^{fl/fl}$ and *Rheb1*$^{Δ/Δ}$ AML mice. (F) Gating strategy for measuring L-GMP frequencies in the spleens of *Rheb1*$^{fl/fl}$ and *Rheb1*$^{Δ/Δ}$ AML mice. (G) L-GMP frequency in the spleen (n = 4). (H) The percentages of AML subpopulations using c-Kit and Gr-1 in the spleen (n = 4). The data are presented as the mean numbers ± SEM.

Figure S3 The function and related signaling pathway of AML cells. (A) GFP^+ cells homing to the BM (left panel) and spleen (right panel), n = 5. (B) The differentially expressed genes of *Rheb1*$^{fl/fl}$ and *Rheb1*$^{Δ/Δ}$ AML cells. (C) Enrichment of M phase mitotic cell cycle-related genes in *Rheb1*$^{fl/fl}$ vs.

Rheb1$^{\Delta/\Delta}$ GFP^{+} cells. NES (normalized enrichment score), and the P values are indicated in each plot. (D and E) The cell cycle status of K^{+}G^{+} (D) and K^{-}G^{+} cells (E) (n = 4). (F and G) The mRNA expression levels of the indicated genes in GFP^{+} (F) and K^{+}G^{-} cells (G). The GFP^{+} cells were sorted, and the RNA was isolated for RT-PCR using the indicated gene primers. The data are presented as the mean numbers ± SEM. (H and I) The normalized MFI of p-S6 (H) and p-4E-BP1 (I) of K^{+}G^{+} and K^{-}G^{+} cells in the BM of both groups (n = 4). The data are presented as the mean numbers ± SEM.

Figure S4 The signaling pathway in human AMLs and murine AML. (A and B) mTOR (A) and 4E-BP1 (B) mRNA expression in human AML cells with t(8;21)(AMLI_ETO), t(15;17)(APL), inv(16)/t(16;16) and t(11q23)/MLL using the HemaExplorer database. The data were analyzed using the Mann-Whitney U test. Significant differences are indicated with asterisks (*, P < 0.05; **, P < 0.01; ***, P < 0.001; ****, P < 0.0001). (C) KEGG analysis of the upregulated pathways in *Rheb1*$^{fl/fl}$ vs. *Rheb1*$^{\Delta/\Delta}$ GFP^{+} cells. (D) KEGG analysis of downregulated pathways in *Rheb1*$^{fl/fl}$ vs. *Rheb1*$^{\Delta/\Delta}$ GFP^{+} cells.

SUPPLEMENTARY METHODS

LSC transplantation

K^+G^- cells (LSCs) were sorted using a BD FACS Aria III flow sorter, and then transplanted into sub-lethally irradiated recipient mice. The survival status was observed and the survival curve was analyzed by Graphpad Prism 6.

Histological and pathological analysis

For pathological analysis, spleen and liver were fixed with 4% formalin, embedded in paraffin, sectioned, and stained with hematoxylin and eosin.

Homing assay

WBMCs were obtained and GFP^+ cells were sorted by Aria III flow cytometer. 1 $\times 10^7$ BM GFP^+ cells were intravenously injected into lethally irradiated (9.5 Gy) recipient mice (CD45.1). The recipient mice were sacrificed to obtain WBMCs and spleen cells after 24 h, and these cells were then labeled for analyzing the number of GFP^+ cells by BD FACS LSRII flow cytometer.

Rapamycin dissolution

Rapamycin (LC Lab) was dissolved in absolute ethanol at 10 mg/ml and stored at -80°C before use. The stock solution was added to the media at a final concentration of 5.45 nM/ml for all the experiments.

Western blotting analysis

BM PMN cells were lysed and their proteins were extracted, separated and electrophoretically transferred to polyvinylidene difluoride membranes (Millipore). The membrane was blocked with 5% non-fat milk for 1 h, washed

for three times, then incubated with the primary antibodies (β-actin（8H10D10）Mouse mAb: CST; Rheb Rabbit antibody: Thermo Fisher) for 1 h at room temperature, and stay overnight at 4℃. After washing for three times the next day, the membrane was incubated with the relevant horseradish peroxidase-conjugated secondary antibodies (anti-rabbit/mouse IgG HRP-linked antibody: CST) for 1 h, then washed for three times. Finally, the reactive proteins were visualized with ImageQuant LAS4000. The density of each band was analyzed with Image J software and normalized with the control actin band.

A

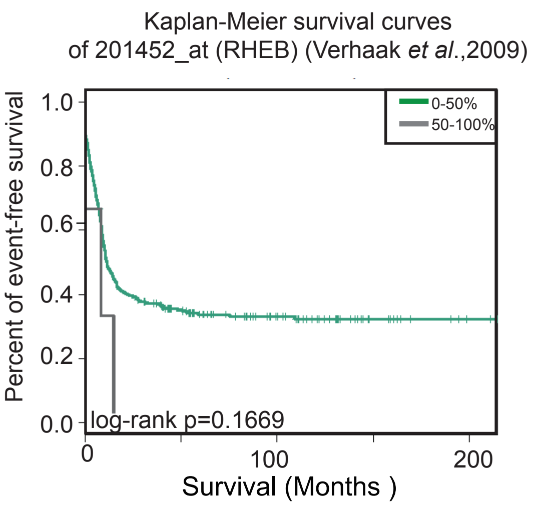

Rheb1 expression	Median survival time
0-50%	11.47
50-100%	8.41

B

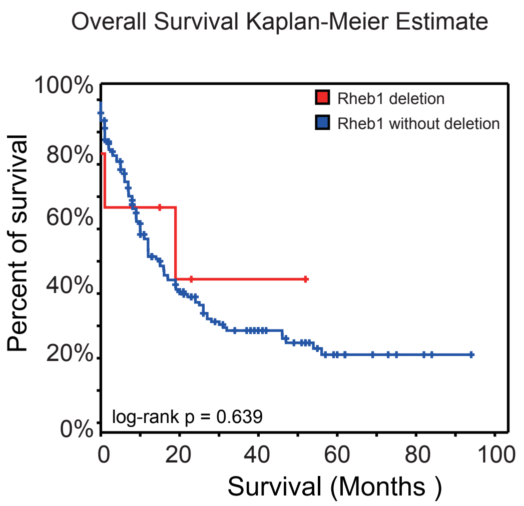

AML (TCGA, Provisional)	#Total cases	#Cases decreased	Median survival (months)
Cases with Rheb1 deletion	6	3	18.96
Cases without Rheb1 deletion	171	111	15.01

Figure S1

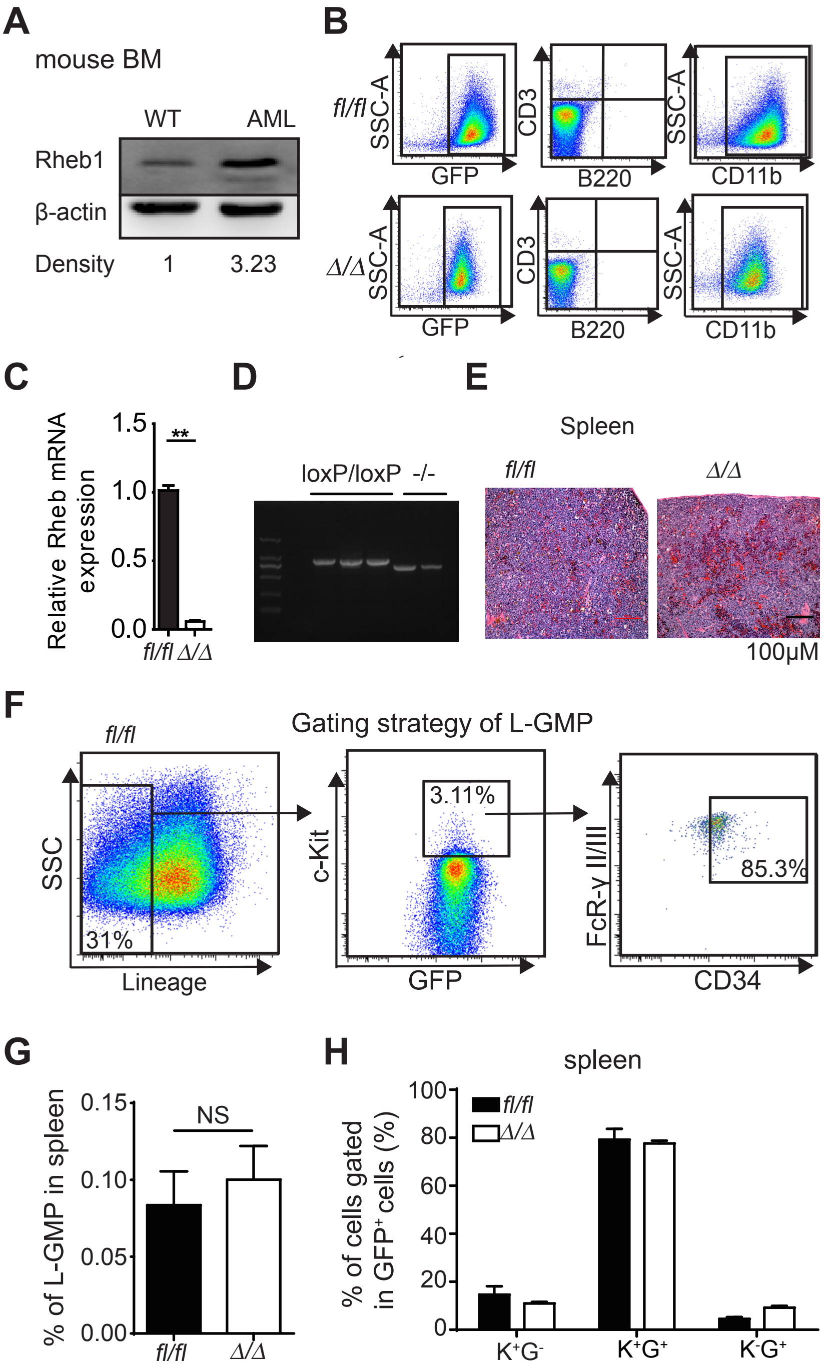
A
mouse BM
WT
AML
Rheb1
β-actin
Density
1
3.23
B
fl/fl
Δ/Δ
SSC-A
GFP
CD3
B220
CD11b
C
Relative Rheb mRNA expression
1.5
1.0
0.5
0.0
**
fl/fl
Δ/Δ
D
loxP/loxP
-/-
E
Spleen
fl/fl
Δ/Δ
100μM
F
Gating strategy of L-GMP
fl/fl
SSC
Lineage
31%
c-Kit
GFP
3.11%
FcR-γ II/III
CD34
85.3%
G
% of L-GMP in spleen
0.15
0.10
0.05
0.00
NS
fl/fl
Δ/Δ
H
spleen
% of cells gated in GFP+ cells (%)
100
80
60
40
20
0
fl/fl
Δ/Δ
K+G-
K+G+
K-G+

Figure S2

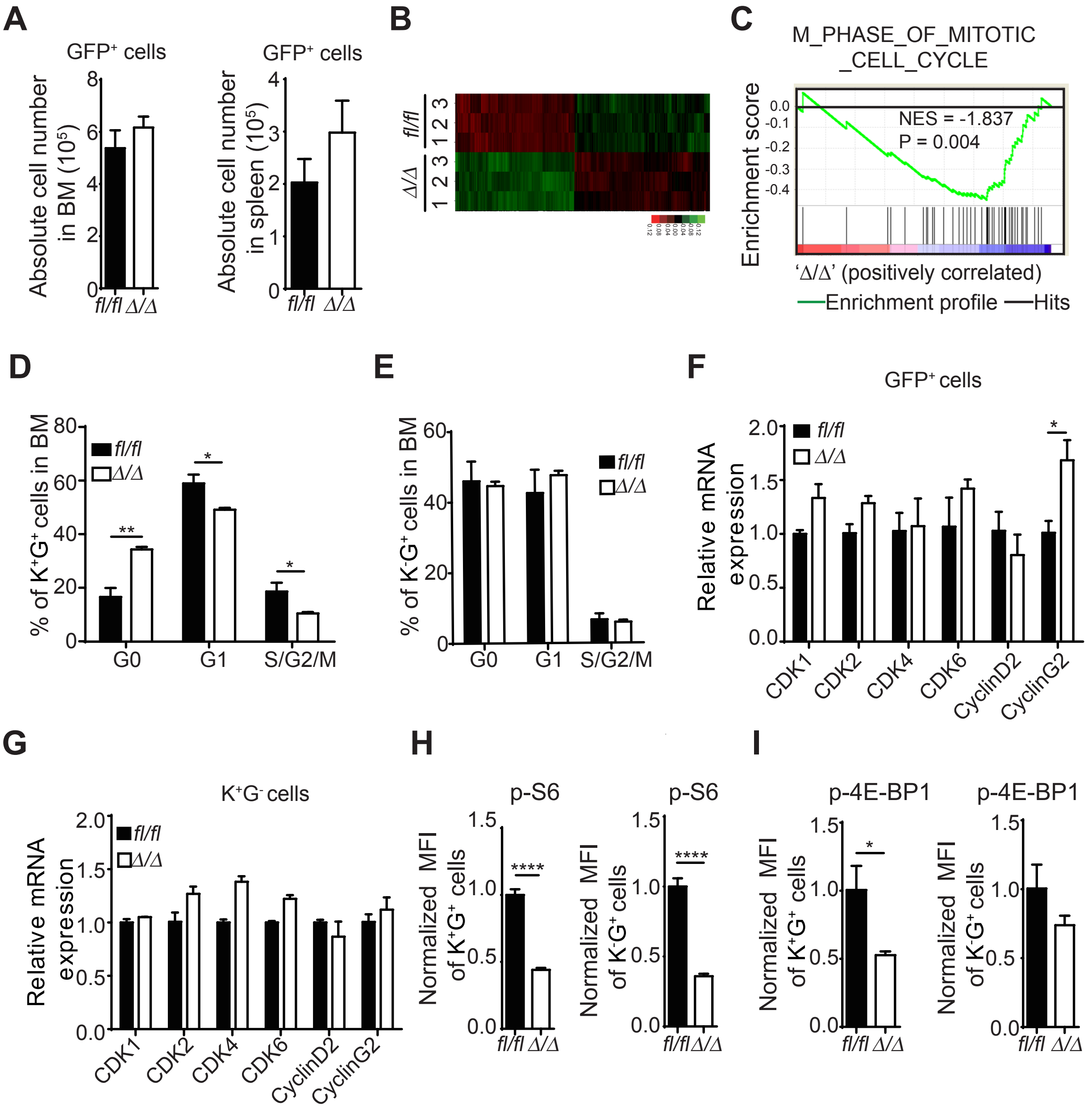

A
GFP⁺ cells
Absolute cell number in BM (10⁵)
fl/fl Δ/Δ
Absolute cell number in spleen (10⁵)
B
fl/fl
Δ/Δ
C
M_PHASE_OF_MITOTIC _CELL_CYCLE
Enrichment score
NES = -1.837
P = 0.004
'Δ/Δ' (positively correlated)
Enrichment profile
Hits
D
% of K⁺G⁺ cells in BM
G0
G1
S/G2/M
E
% of K⁻G⁺ cells in BM
F
GFP⁺ cells
Relative mRNA expression
CDK1
CDK2
CDK4
CDK6
CyclinD2
CyclinG2
G
K⁺G⁻ cells
H
p-S6
Normalized MFI of K⁺G⁺ cells
Normalized MFI of K⁻G⁺ cells
I
p-4E-BP1

Figure S3

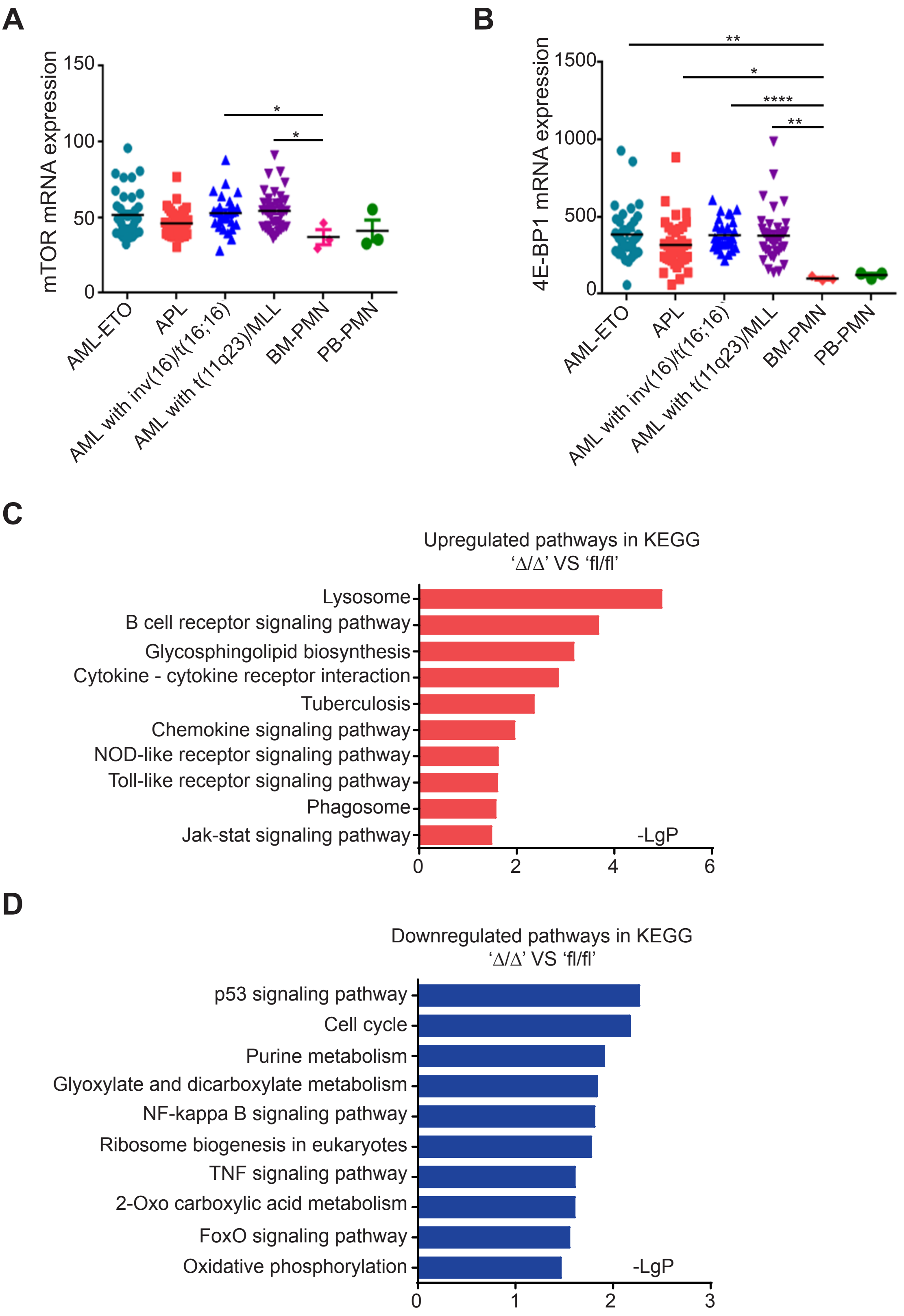
A
mTOR mRNA expression
150
100
50
0
*
*
AML-ETO
APL
AML with inv(16)/t(16;16)
AML with t(11q23)/MLL
BM-PMN
PB-PMN
B
4E-BP1 mRNA expression
1500
1000
500
0
**
*
****
**
AML-ETO
APL
AML with inv(16)/t(16;16)
AML with t(11q23)/MLL
BM-PMN
PB-PMN
C
Upregulated pathways in KEGG
'Δ/Δ' VS 'fl/fl'
Lysosome
B cell receptor signaling pathway
Glycosphingolipid biosynthesis
Cytokine - cytokine receptor interaction
Tuberculosis
Chemokine signaling pathway
NOD-like receptor signaling pathway
Toll-like receptor signaling pathway
Phagosome
Jak-stat signaling pathway
-LgP
0
2
4
6
D
Downregulated pathways in KEGG
'Δ/Δ' VS 'fl/fl'
p53 signaling pathway
Cell cycle
Purine metabolism
Glyoxylate and dicarboxylate metabolism
NF-kappa B signaling pathway
Ribosome biogenesis in eukaryotes
TNF signaling pathway
2-Oxo carboxylic acid metabolism
FoxO signaling pathway
Oxidative phosphorylation
-LgP
0
1
2
3

Figure S4

Table S1 Primer sequence list

Primer	Sequence（5'-3'）	
	**mouse**	
	**Forward**	**Reverse**
**GAPDH**	TGTGTCCGTCGTGGATCTGA	TTGCTGTTGAAGTCGCAGGA
**Rheb1**	GTTCACCAAGTTGATCACGGT	ATATTCATCCTGCCCCGCTG
**BAX**	TGGAGCTGCAGAGGATGATTG	AGCTGCCACCCGGAAGA
**Bim**	TTGGAGCTCTGCGGTCCTT	CAGCGGAGGTGGTGTGAAT
**Puma**	GCGGCGGAGACAAGAAGA	AGTCCCATGAAGAGATTGTACATG
**P53**	AAGATCCGCGGGCGTAA	CATCCTTTAACTCTAAGGCCTCATT
**Mcl-1**	CCCTCCCCCATCCTAATCAG	AGTAACAATGGAAAGCATGCCAAT
**P18**	TACGAATTCATGGCCGAG	TGGCTCGAGTCACTGCAGGCTT
	CCTTGGGGGA	GTGGCT
**P19**	AGGCCGGCAAATGATCATAGA	GGTGGATACCGGTGGACTGTG
**P21**	AATCCTGGTGATGTCCGACC	TTGCAGAAGACCAATCTGCG
**P27**	TATGGAAGAAGCGAGTCAGC	GCGAAGAAGAATCTTCTGCAG
**P57**	CAGCGGACGATGGAAGAACT	CTACGCAACCATCTCCGGTT
**CDK1**	CAAAGCTGGCTGGGTTTCAC	TCTGACCATGCAGAGCACAG
**CDK2**	AGGTGGAGAAGATTGGAGAGG	CCCGTCAACTTGTTTTTGGCT
**CDK4**	CCCTCTTCTCACTCTGCGTC	TGCCAGAGATGGAGGAGTCT
**CDK6**	TGACACTGTGCACACATCAAA	AGACCAGTGAGGAGGGCAT

**CyclinG2**	AGGGGTTCAGCTTTTCGGATT	AGTGTTATCATTCTCCGGGGTAG
**CyclinD2**	GAGTGGGAACTGGTAGTGTTG	CGCACAGAGCGATGAAGGT
**SLC7A7**	CCACACCCATAACGCTGAGT	TGCCCTCTAGCAAACACAGG
**CD163**	TGCCTCTGCTGTCACTAACG	TTCATTCATGCTCCAGCCGT
**TGFβ1**	CTGCTGACCCCCACTGATAC	AGCCCTGTATTCCGTCTCCT
**MMP8**	AGCGCTTCTTCAGCTTAGCA	GTCCACTTGGGACTTCCTGG
**LTF**	TCCCGAAGCACGAATGACAA	GGGAGGCAAAGAGCTGGAAT
**HOXA9**	CCCCGACTTCAGTCCTTGC	GATGCACGTAGGGGTGGTG
**MEIS1**	CATGATAGACCAGTCCAACCGA	ATTGGCTGTCCATCAGGGTTA
**Primer**	**human**	
	**Forward**	**Reverse**
**GAPDH**	GGTCGTATTGGGCGCCTGGTC	TGACGGTGCCATGGAATTTGCCA
**Rheb1**	AGGGTCATGACGCAGCGAGT	TGCGTCGGGGCGACGTTTTA
